# Impact of Printing Orientation on the Accuracy of Additively Fabricated Denture Base Materials: A Systematic Review

**DOI:** 10.3390/dj12070230

**Published:** 2024-07-22

**Authors:** Maram A. AlGhamdi, Mohammed M. Gad

**Affiliations:** Department of Substitutive Dental Sciences, College of Dentistry, Imam Abdulrahman Bin Faisal University, P.O. Box 1982, Dammam 31441, Saudi Arabia; maalghamdi@iau.edu.sa

**Keywords:** 3D printing, orientations, dental prosthesis, accuracy

## Abstract

Printing orientation is one of the printing parameters that affect the properties of three-dimensional (3D)-printed resins. Different printing orientations and directions have been suggested; however, no clear and specific orientations are recommended in the literature in terms of the printing orientation effect on the accuracy and fit of 3D-printed removable dental prostheses. This review aimed to evaluate the effect of printing orientation on the fit and accuracy of 3D-printed removable dental prostheses. The PubMed, Scopus, and Web of Science databases were searched for published articles that investigated the effect of printing orientations on the accuracy and fit of the 3D-printed denture base. Full-length English published articles were searched between January 2010 and December 2023, which examined topics related to printing orientations, building angles, 3D printing, printing technology, accuracy, dimensional changes, internal fit, marginal integrity, marginal discrepancies, trueness, precision, and adaptation. Of the ten included studies, one investigated maxillary and mandibular denture bases, seven assessed maxillary denture bases, and two evaluated mandibular bases. Different printing orientations, ranging from 0° to 315°, were explored, with a higher prevalence of 0°, 45°, and 90°. The included studies utilized stereolithography and digital light processing printing technologies. High accuracy was observed at 45°, followed by 90. Additional struts and bars on the cameo surface increased the accuracy of the 3D-printed denture base. These results shows that printing orientation has a significant effect on the accuracy of 3D-printed resin, with 45° exhibiting the highest accuracy. In addition to the support structure, the density and position can impact the accuracy.

## 1. Introduction

Removable dental prosthesis is the preferred initial treatment for edentulous individuals to restore phonation, esthetics, and mastication [1]. The fitting between the denture base and the tissue underneath results in denture stability, support, and retention and is significantly influenced by denture processing [2]. For many years, the traditional approach to denture processing, known as compression molding, has yielded positive clinical outcomes. Nevertheless, employing this method has the disadvantage of requiring numerous clinical and laboratory steps and time-consuming procedures [3]. Polymethyl methacrylate (PMMA)-based dentures can now be produced in a simple digital workflow thanks to recent advancements in computer-aided design/computer-aided manufacturing (CAD/CAM) techniques in dentistry [2,3].

There have been recent reports on the use of digital technology, such as CAD/CAM systems, in removable dental prostheses’ fabrication [4,5]. Based on the fabrication method, two methods were reported for CAD/CAM denture fabrication: subtractive (SM) and additive (AM) [2,6]. SM involves milling a pre-polymerized PMMA resin block to achieve the desired shape. In contrast, AM or three-dimensional (3D) printed denture parts are fabricated through layer-by-layer printing using photopolymer resins [2,5,6,7]. Additive manufacturing (AM) offers advantages over subtractive manufacturing (SM) such as reduced material waste, cost-effectiveness, and shorter printing times [8].

The three main stages in the AM process for manufacturing complete denture bases include data processing, printing, and post-printing procedures [6]. In each stage, there are parameters that affect the properties of the printed object [9,10]. For pre-printing, the photopolymerized resin type, compositions, and photoinitiator have roles in the polymerization process of 3D-printed resin [6,9]. In the printing setup and printing process, the parameters include the printing layer thickness, printing orientations, support (structure, density, and position), printing speed, and light penetration depth, as well as the printing technology [10,11]. The printed object is in a green state and additional polymerization cycles are required to increase the degree of monomer conversion and decrease the residual monomer content; therefore, post-printing polymerization is suggested [10,12]. The post-printing polymerization process includes different variables such as cleaning methods, polymerization time, and temperature, as well as curing machine, light type, and wavelength [13]. Notably, several workflow parameters, such as the printing layer thickness, build orientation, slicing and support structures, and post-processing conditions (rinsing solutions, post-polymerization duration, and temperature) can affect the accuracy of a printed object [9,10,14]. Therefore, obtaining superior printing outcomes for clinical applications requires improving the AM workflow’s parameters. An essential data processing component is setting the build orientation [15]. Different printing orientations have been suggested and have shown clinical significance, with greater variations between the same orientations in different studies [16].

Reproducing a denture shape designed using CAD with the uppermost accuracy is crucial for the denture to fit precisely to the patient’s mouth [17]. Both trueness (closeness of measured values to the true value) and precision (closeness of measured values throughout repeated measurements) are defined by the International Standards Organization (ISO, 5725-1) as accuracy. Trueness refer to the degree of agreement between the mean arithmetic of the variety of the testing results and the agreed reference value, which displays the discrepancy between the desired dimensions (reference value) and the dimensions of printed objects. Precision is defined as the closeness of the agreement test results in which the printed objects have the same dimensions, demonstrating the ability of repeated manufacturing with exactly the same dimensions [18,19].

Different printing mechanisms, printing materials, and geometries of the denture base have a significant impact on accuracy [20,21,22,23]. Therefore, the accuracy may depend on both the component orientation and component geometry [9]. Changing printing orientations leads to variations in other variables such as support structures and position (which directly affect accuracy), starting points, layer direction, light source, direction of the printer platform movement, and unreactive monomer sagging [24,25]. With different printing orientations, the interaction between the aforementioned factors can improve or impair the denture base accuracy in terms of the separation effect force and polarization shrinkage of the printed denture [17]. Therefore, CD bases fabricated within the appropriate build orientations with a positive interaction of these factors are more accurate, and optimizing build orientation may help to lessen pressure points and increase denture retention and mucosal support. To date, no optimal printing orientation for achieving highly accurate printed dentures has been established. Therefore, this review aimed to determine the optimum printing orientation for printed dentures with high accuracy.

## 2. Materials and Methods

The Preferred Reporting Items for Systematic Reviews and Meta-Analyses (PRISMA) guidelines were used to conduct this review. The study was designed to answer the following question: “What is the effect of printing orientation on the accuracy and fit of a 3D-printed denture base?” based on the PICO search strategy. Electronic searches were performed in three databases (PubMed, Web of Science, and Scopus) within the search period between January 2010 and December 2023, using keywords (Table 1). The articles were reviewed according to the inclusion and exclusion criteria.

### 2.1. Inclusion and Exclusion Criteria

Studies were included if they investigated the effect of different printing orientations on the accuracy of 3D-printed denture bases using denture base resins. Studies were excluded if they focused on materials other than denture base resins or if they did not evaluate accuracy metrics. All the printed resins with denture configurations were included, and their dimensional changes, trueness, precision, adaptation, and fit were also assessed. In addition to full-length articles, in vitro and English-language articles were included. Moreover, any study that investigated specimens rather than denture configurations was excluded. In addition to articles irrelevant to the focus question, case report studies, abstracts, languages other than English, reviews, and short communications were excluded.

### 2.2. Study Selection and Data Extraction

After duplicate studies were deleted, two authors (M.A. and M.M.G.) independently reviewed the abstracts of the searched articles according to the keywords and inclusion criteria. In case of disagreement, the two authors discussed the point of difference until a consensus was reached. Data were collected in an Excel spreadsheet (Table 2) with the required headlines and items for analysis.

### 2.3. Quality Assessment of Included Studies

The modified consolidated standards of reporting trails (CONSORT) guidelines for in vitro studies were used to rate the included studies’ quality [26]. The tool contains 7 domains listed in Table 3, presented as “yes” or “no” to assess and evaluate each article. Two investigators (M.A.A. and M.M.G.) evaluated the quality of the studies and assessed the risk of bias using previously reported tools independently. Table 3 shows the items used to assess the quality of each study. Per article, each parameter received “yes” if the parameter was clearly presented and received “no” in the absence of parameter information. According to the “yes” and “no” score, each study estimated the risk of bias of the study: low, medium, or high (Table 3 footnotes).

**Table 2:** Included studies and printing parameters with accuracy outcomes per study.

Author/ Year/ Specimens	Printers and Technology	Specimens Configuration/ Sample Size	Orientations	Layer Thickness	Supports Position	Post-Curing Conditions	Scanners	Measurement Method/Unit	Reference Model	Software	Main Finding and Recommendations
Jin et al., 2018 [27]	NextDent Base; NextDent Printer (Bio3D W11; NextDent) DLP	*Maxillary and mandibular denture base*/with tooth sockets and without teeth (n = 10) N = 40	90°, 100°, 135°, 150°	100 µm	Cameo surfaces	LC 3DPrint Box; NextDent 15 min Temp. According to manufacturer instructions (no info)	Lab scanner (Identica Blue T500; Medit) used to detect 10 µm differences	Superimposed/ RMSE, PA, and NA/mm	Reference cast	Geomagic Control X; 3D Systems	No significant effect of printing angle on tissue surface adaptations
Hada et al. 2020 [28]	Clear resin; Formlabs Printer: Form 2; Formlabs SLA	*Maxillary denture base*/with tooth sockets and *without teeth* (n = 6) N = 18	0°, 45°, 90°	100 µm	Cameo and intaglio surface	(Form Cure; Formlabs) 60 C for 10 min	Lab scanner 3D optical scanner (NeWay; Open Technologies, Rezzato, Italy)	Superimposed with a best-fit alignment RMSE (mm)	Master data	3D analysis software (CATIA V5; Dassault Systèmes, Vélizy-Villacoublay, France).	3D-printing accuracy is angle-dependent and 45° showed the highest accuracy
Yoshidomea et al. 2021 [29]	DENTCA Denture Base II Two printers SLA and one printer DLP	*Maxillary denture base*/without tooth sockets and without teeth (n = 5) N = 40	0°, 45°, 90°, 135°, 180°, 225°, 270°, 315°	100 µm	Cameo and intaglio surface	(HiLite Power 3D, Kulzer, Hanau, Germany) 15 min Temp. NS	Lab scanner (R700, 3Shape, Copenhagen, Denmark)	Superimposed/with a best-fit alignment RMS (mm)	Master model (wax denture base scanned)	Geomagic Design X, 3D Systems, Rock Hill, CA, USA	45° showed the highest accuracy. Support structures and built pitch affect the accuracy
Cameron et al., 2022 [30]	3D+; NextDent Printer: (NextDent 5100) DLP	*Maxillary denture base*/with tooth sockets and without teeth (n = 10) N = 70	0°, 15°, 45°, 60°, 90°	50 µm	Cameo and intaglio surface + support struts on both surfaces	30 min (LC-3D Print Box; NextDent B.V.) 30 min Temp. MR	Lab scanner desktop laser scanner (E3; 3Shape A/S)	Superimposed/with a best-fit alignment RMSE (µm)	Reference cast	Geomagic Control X v20.0; 3D Systems Inc.	Maximum trueness found with 45, 60, and 90°. Also, the support strut affected the level of trueness
Charoenphol et al. 2022 [31]	Optiprint Gingiva, Dentona, Dortmund, Germany printer: Asiga Max, Asiga, DLP	*Maxillary denture base*/without tooth sockets and without teeth (n = 10) N = 30	0°, 45°, 90°	100 µm	Cameo surface	Asiga Flush 30 min Temp. MR	Lab scanner Extra-oral scanner (E4 scanner, 3 Shape Dental System)	Surface-matching software superimposed/with a best-fit alignment RMSE (mm)	Reference cast	Geomagic Design X, 3D Systems, Rock Hill, CA, USA	The printing angle had no significant effect of the overall accuracy
Song et al. 2023 [32]	DENTCA base material Printer: (Pro95, SprintRay) DLP	*Maxillary denture base*/with tooth sockets and without teeth (n = 5) N = 70	0°, labial 45°, labial 90°, posterior 45°, posterior 90°, buccal 45°, buccal 90°	50 µm; 100 µm	Cameo surface and alveolar sockets	(ProCure, Sprintray, USA) 40 min Temp. 60 °C	Laboratory scanner (Ceramill Map 600, Amann Girrbach, Austria)	Superimposed/with a best-fit alignment RMS (mm)	Reference cast by “N-Point Alignment” and “Best Fit Alignment”	Geomagic Wrap, 3D Systems, USA	Build orientation affect the accuracy and 45° and 90° showed the satisfactory accuracy
Lee et al. 2023 [33]	NextDent Denture 3D+ Printer: (Max UV; Asiga) DLP	*Maxillary denture base*/with tooth sockets and without teeth (n = 10) N = 120	0°, 45°, 90°	50 µm; 100 µm	Cameo surface	(Cure M U102H; Graphy) low-viscosity 5 min high-viscosity 15min Temp. NS	Laboratory scanner (Medit T710; Medit)	Superimposed/ best-fit alignment RMS (µm)	Reference CAD data	Geomagic Control X; 3D Systems	The highest trueness was found with 45°
Gao et al. 2021 [34]	VisJet M3 crystal Multijet Printer: (ProJet MJP 3600 Dental MJP	*Mandibular* *denture bases/with teeth* (n = 9) N = 27	0°, 45°, 90°	16 µm	Cameo surface	NS 30 min 158 °C	Lab scanner Optical surface scanner (Activity 880, Smart Optics, Bochum, Germany)	Superimposed/ best-fit alignment (RMS) (mm)	Reference STL files	Geomagic Wrap 2015 software, 3D Systems	The 45° build orientation showed higher accuracy
Chaiamornsup et al., 2023 [35]	(Dima Print denture base) Printer: Cara Print 4.0; Kulzer GmbH DLP	*Mandibular* *denture bases*/with tooth sockets and without teeth (n = 6) 16 DESIGN N = 96??	0°, 45°, 90°, 135°, 180°, 225°, 270°, 315°	50 µm	Cameo surface + transverse bar	(HiLite Power 3D; Kulzer GmbH) 10 min Temp. NS	Laboratory scanner (D2000; 3Shape, Copenhagen, Denmark) with 5 µm accuracy	Superimposed/ best-fit alignment RMS (mm)	Original CAD	FreeForm ModelingPlus V12.0; Geomagic, NC, USA	The 270° build orientation is recommended
Unkovskiy et al., 2021 [36]	Denture base OP Formlabs, Printer: Form 2; Formlabs SLA	*Maxillary denture base*/with tooth sockets and without teeth (n = 5)	0, 45, and 90	NS	Cameo surface	FormCure, Formlabs, 80 °C for 60 min	Lab scanner D2000, 3Shape, Copenhagen, Denmark	Superimposed/ best-fit alignment RMSE, PA, and NA (mm)	Reference cast	Geomagic Control X, 3D systems	The 90 degree build angle may provide the best trueness. Higher precision was revealed in the DLP
	V-print Dentbase–VOCO Printer: Solex 350 PLUS, DLP					LC-3DPrint Box, 3D Systems 30 min Temp. MR					

NS, not stated; RMSE, root mean square error; PA, positive average; NA, negative average; MR, manufacturer recommendation; SLA, stereolithography; DLP, digital light processing printing; MJP, multijet printing.

### 2.4. Quantitative Assessment of Included Studies

Table 4 summarizes the main values and standard deviation (SD) of the results of all included studies. The collected data were evaluated for eligibility for the meta-analysis. The collected studies were categorized as maxillary and mandibular denture studies, and when data were available and eligible, a meta-analysis was performed.

A meta-analysis could not be conducted due to the substantial variations among the included studies. These variations included the denture type (maxillary, mandibular, or both) and different printing orientations and directions in addition to other variables such as such as the printing layer thickness (16, 50, 100 µm, or not stated), resin type, and viscosity, the variation in printing technology (SLA, DLP, or MJP), and post-curing conditions (machines, time, and temperature). Furthermore, variations in the support structure conditions (density and position) and the inclusion of a transverse bar of contact with the denture base at different surfaces and different points. This is in addition to the different aging and immersion time, which all further contributed to the complexity of the meta-analysis conduction. The aforementioned variations between included studies make the quantitative meta-analysis non-eligible; thus, a qualitative descriptive analysis was performed.

## 3. Results

### 3.1. Search and Selection

**Figure 1:**
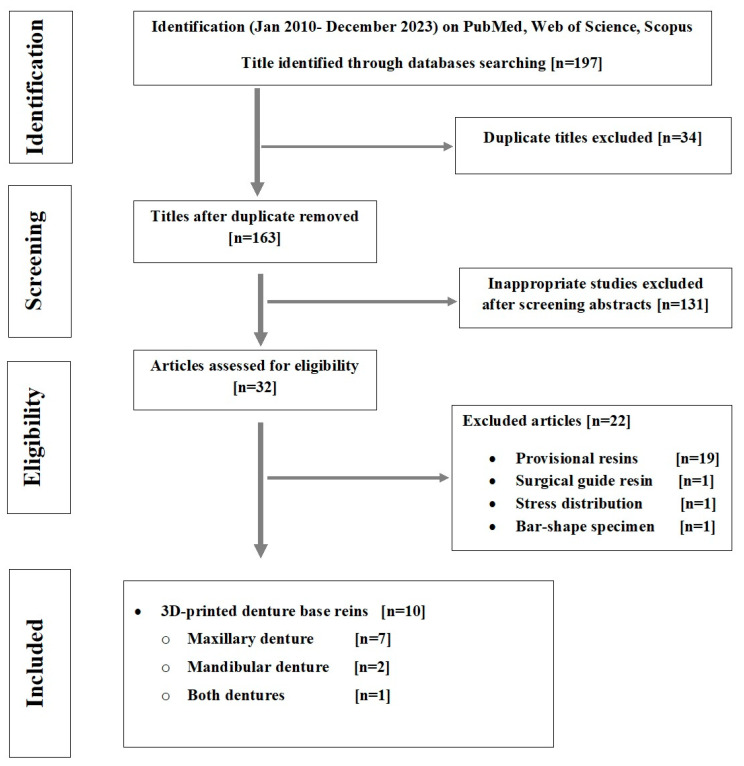
PRISMA flow chart of the study selection process.

Following database screening and duplicate removal, 163 articles were found (Figure 1, Appendix A). Following a title screening, only 32 articles remained. Further exclusions were made after examining abstracts. The full texts of 32 studies were reviewed and, after merging hand-searched articles, only 10 met the inclusion criteria.

### 3.2. Risk of Bias Finding

The quality of the included studies was assessed using a modified CONSORT checklist (Table 3) [26]. None of the included studies reported sample randomization and there was no blinding, while four studies reported sample size calculation, resulting in eight studies with medium risk and two studies with high risk. All studies clearly described details about the printing workflow and printing orientation design, slicing, and different measurement methods. Additionally, all the studies contained adequate reports on each element of the manuscript structure (background, objectives, interventions, and outcomes).

### 3.3. Main Results

Out of 197 studies, 10 [27,28,29,30,31,32,33,34,35,36] were included in this systematic review. Of the ten included studies, the accuracy of 3D-printed denture base resins was evaluated: one study assessed both the maxillary and mandibular bases [27], seven evaluated the maxillary base [28,29,30,31,32,33,36], and two assessed the mandibular base [34,36]. Regarding printing technology, digital light processing (DLP) and stereolithography (SLA) were distributed among the included studies, with the prevalence of DLP in six studies [27,30,31,32,33,34,35], SLA in one study [28], and two studies that compared both technologies [29,36] in addition to one study which investigated multijet modeling printing (MJP) technology [34].

The 3D digital superimposition on the master data was carried out as an evaluation method using Geomagic in all included studies, except for one study that used CATIA V5 [28] software. The root mean square/root mean square error (RMS/RMSE) was the measurement method in millimeters (mm) in seven studies [27,28,29,32,34,35,36], whereas the other three studies [30,31,33] measured in micrometers (µm), which were converted to mm to standardize the accuracy values. Two common printing layer thicknesses were employed, which were 50 µm [30,35] and 100 µm [27,28,29,31], and some studies compared both thicknesses [32,33]. One study used a 16 µm multijet 3D printer [34], and in one study, the layer thickness was not specified [36]. All dentures were post-cured according to the manufacturer’s recommendations; however, some studies [29,32,33,36] used third parties (different machines rather than the 3D systems recommended by the manufacturer). Additionally, the processing time varied from 10 to 30 min, and the temperature ranged from 40 to 60 °C, with one study using a high temperature of 158 °C [34]. Different brands of laboratory (desktop) scanners were used in all included studies for printed denture scanning.

**Figure 2:**
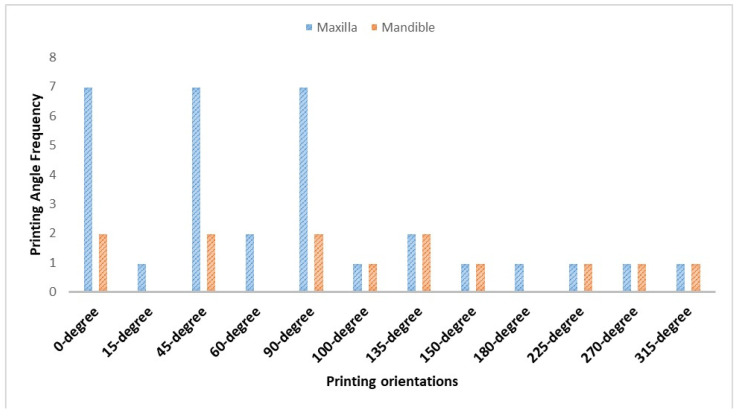
Frequency of printing angles of included studies.

Different printing orientations (0–315°) were investigated (Figure 2), with a higher prevalence of 0°, 45°, and 90°. For the printing angle, comparisons of 0°, 45°, and 90° were investigated in five studies [28,31,33,34,36], whereas other studies added orientations ranging from 0 to 90° [30,31], up to 150° [27], with some angles increasing to 315° [29,35]. Different measurement areas with different printing angles were suggested and evaluated in the included studies. Overall, intaglio surface adaptation, trueness, and precision were evaluated in almost all included studies, whereas peripheral and posterior palatal seal areas and primary stress-bearing areas were evaluated in one study [31]. The density and position of supports varied according to the printing orientation. Support positions were designed on the cameo surface [27,31,36] or on both cameo and intaglio surfaces [28,29,30], with one study utilizing additional supports at the cameo and intaglio [30] and another adding support bars between the lingual flanges of the mandible [35].

For the maxillary denture base, all included studies reported that 45° showed the highest accuracy compared to other printing angles [28,29,30,32,33], followed by 90° [30,32], except two; however, other studies showed non-significant differences in accuracy between printing angles [27,31]. For the mandible, one study reported the highest accuracy at 45° [34], and another reported a printing angle of 270° [35]. The color map deviation confirmed all findings (45° accuracy) regarding +ve, −ve, and RMSE, with some exceptions. One study [27] found the highest accuracy at 135° for the maxilla and 100° for the mandible based on a color map display. Some studies [28,29,30] reported additional factors associated with the printing angle, which were related to the supporting structures (density, size, and position). Therefore, the angle between the printing direction and the build platform changed, and the support structures were adjusted accordingly [27,28,29,30,31,32,33,34,35,36]. Another study also highlighted the influence of the starting printing point on accuracy, as it was determined by the support position of the printing angle [29,35]. In addition to support, one study [35] added a transverse bar connecting the lingual flanges (right to left) of the mandibular dentures as a method to decrease the error rate and increase the accuracy.

The position of the support changed according to the orientation and either automated design with the orientations or manually designed if additional supports were required [10]. Most of the studies [27,31,32,33,34,35,36] designed the support at the cameo surface, while other studies [28,29,30] designed to be at both the cameo and intaglio surface. All of the included studies placed support away from the tooth socket except one study [32]. Not only did the support structure and position affect the accuracy, but the method of support removal also had an effect. Two studies [29,30] focused on the effect of the support position and its effects on the accuracy.

As the post-curing has an impact on the properties of printed resins, some studies followed the manufacturer’s recommendations and used their own post-curing unit with the recommended time and temperature [27,28,30,31,36], while other studies used a third-party unit with different times and temperatures [29,32,33,35] and one study did not mention the curing machine type [34]. The post-curing times investigated were 10, 15, 30, 40, and 60 min. The variation between times revealed that the orientation has a greater impact on the accuracy. At 10 min, 45 degrees showed the optimum accuracy [28,35]. At 15 min, one study showed no significant difference [27], while another study [29] recommended 45 degrees. At 30 min, there was no significant difference [31], while 45 degrees showed the maximum accuracy [30,34]. At 40 min, 45 and 90 degrees showed the highest accuracy [32]. One study used two curing machines with different post-curing times (30 and 60 min) and found that 90 degrees showed the best accuracy [36].

## 4. Discussion

### 4.1. Clinically Acceptable Value of Accuracy

Denture base accuracy is directly related to the gap between the intaglio surface and the denture foundation tissue. A higher accuracy results in a smaller gap, leading to an increased retention of CDs [35]. To assess accuracy from a clinical point of view, there was no clear agreement about the clinically acceptable value that the denture could be compared to [17]. Tissue compressibility plays a role in determining the clinically acceptable values for denture accuracy and adaptation [37]. Recent studies found that the average deviation considered acceptable is 0.03 mm [28,31,38]. After the CD insertion, under maximum force the mucosal thickness approximately decreased up to 0.3 mm [35,37,38], while another study [39] reported that the oral tissue can be compressed to 0.0375–0.05 mm, resulting in a better fit between the base and the mucosa, ultimately ensuring effective border sealing [35,37,39]. If the deviation is less than this range, the adaptability is within a clinically acceptable value [37]. Owing to patient differences in tissue compressibility, the range of clinically acceptable values may be taken into consideration, rather than the precise value. Another factor was the variation in compressing in the same jaw beyond the compressibility in local areas, resulting in pain upon pressure, discomfort, and/or loss of retention with clinical use. Therefore, 0.03 mm–0.05 mm is the range within which the average deviation is clinically acceptable, whereas deviations beyond this average are not clinically acceptable.

### 4.2. Printing Technology

SLA and DLP are the most common technologies used for denture base printing, while a recent study has investigated a technology called multijet modeling printing (MJP) [40,41]. SLA utilizes dynamic writing with a condensed laser beam, while DLP uses digital micromirror devices to project ultraviolet (UV)-layered images onto a selected part of the entire x/y. MJP uses several nozzles to jet one or more liquid photopolymers onto a building platform [42]. SLA is being compared with DLP, while no comparison with MJP is introduced for denture printing with different printing orientations. Despite the high laser scanning velocities used in SLA, DLP can simultaneously light-polymerize all portions of a given slice, thus significantly speeding up 3D-printing times between layers and reducing the printing time [9,30,31,32,33,34,43]. During printing, printed objects are hung upside down on the build platforms in the SLA and DLP printers, which are located at the top of the printers [34]. The build platform of the MJP printer is located at the bottom of the printer, in contrast to SLA and DLP printers, and the object is manufactured on top of the platform. MJP offers some advantages such as a print object with high resolution (0.010 mm), the immediate polymerization of printed resin, being less time-consuming in the post-curing process, and having the capability to utilize different printed resins in a single printing order [34,44]. Although MJP has several advantages, only one study used MJP which necessitate further investigation utilizing MJP technology.

Three printing axes (X, Y, and Z) are designed for 3D-printed objects. The accuracy of the printed object implies that the reproducibility differs among the printing axes [27,29]. By changing the orientation, the printed object changes regarding the printing axes, which results in a change in the light source, light penetration depth to cure the monomer, and layer directions [27,28,29,30,35,36]. The accuracy is affected by the light penetration depth, laser intensity, and speed, and all these changes in the layer direction and area to be cured change with each orientation, finally affecting the reproducibility and accuracy [28,32]. There is a relation between light refraction and the printing axes, as the light refraction toward X and Y is more than that towards the *Z*-axis. The printing length in the 90° orientation along the vertical axis showed more reproducibility and consequently a high accuracy, followed by a high accuracy with 45° as the denture configuration, making the horizontal part of the palate approximately come along with the vertical axis [30,33,34].

### 4.3. Denture Base Scanning, Accuracy Measurement Methods, Unit, and Evaluation Criteria in Relation to the Printing Angle

All of the included studies used lab scanners for scanning the printed denture bases. In addition to the standardization when using lab scanners in in vitro studies, it was claimed that the scanning procedure using the laboratory scanner for denature adaptation was deemed to be adequately accurate [16,20,27,29,30,36]. Even so, the measurement should be performed by using Coordinate Measuring Machines (CMMs) which represent an accurate and traceable standard method for linear and volumetric metrological measurement [45,46,47,48,49]. Three-dimensional superimposition analysis is more accurate than conventional manual measurement techniques [17,35,36]. The degree and location of the dimensional changes that take place during denture manufacturing have been evaluated using a variety of methods, with advanced two-dimensional (2D) and 3D measurements among them. Measuring denture base adaptations has recently become more popular with the use of extraoral scanners and surface-matching software [50]. The accuracy and adaptability of the fabricated prostheses are measured using manual or digital methods. The advantage of the digital method is that it avoids human and manual errors, allowing measurement at any selected point [17,50]. One digital method is the 3D superimposition method, which is used in all of the included studies [27,28,29,30,31,32,33,34,35,36]. In this method, all steps, from scanning and alignment to superimposition analysis, are performed using computer software. Additionally, the digital model provides an opportunity to select all the required points and calculate all the points reflecting the deviations [29]. RMSEs and color maps that compare the digitally superimposed distances between the reference and produced denture bases have become popular multidimensional metrics using computer technology [20,34]. Adaptation is obtained using the RMSE, which involves dividing the sum of all absolute values of the deviations, representing the distances between the point clouds of the reference model [31]. Greater error is indicated by higher RMSE values, which represent the variations in characteristics between the produced and reference dentures [20,34].

The features of AM dental devices permit the fabrication of CDs with specific geometry and allow the production of more accurate CD bases [9]. The edentulous maxilla’s anatomical and histological features show that various parts require different levels of tolerance to deviations: a specific relief (negative deviation) is needed for the incisive papilla, torus palatinus, and median palatine suture areas, while in the posterior palatal seal area, for example, greater adaptation and pressure (impingement, positive deviation) within the physiological limit are needed to improve retention, lessen the gag reflex, and prevent the entry and accumulation of food and debris [32]. Therefore, building color maps and segmenting the intaglio surface into regions is necessary to evaluate region-specific misfits [32]. The color map displayed the surface-matching differences between the reference and fabricated dentures [35]. The color map revealed that the printing orientation changes the position of the positive and negative deviations, which can be used in the future as a guide for relief and pressure areas [29,32].

In the 0° group, there was a higher chance of mucosal pressure pain. While the 45° and 90° groups displayed negative deviations (interspace) in the posterior palatal seal area. It is possible that these individuals did not have adequate border sealing, which led to poor denture retention. However, the base’s deviation distributions with build orientations of 45° and 90° may help to keep the denture balanced when the occlusal force is applied. In addition, the distribution of deviation in the 45° and 90° orientations resulted in a favorable fit (slight positive deviation at the posterior palate and slight negative deviation at the relief areas) making these orientations more harmonious with the anatomical topographies of the edentulous maxilla [32,33].

### 4.4. Factors Affecting Accuracy and Their Interrelationship

Many factors influence the effect of build angle changing with the platform during printing on the accuracy of a 3D-printed denture base, such as the support structures, printing layer thickness [29,31,32], exposure time, liquid resin type, light penetration depth, separation force [16], method and time of removal of the support structure, and the starting points of the build, which change with variations in the build angle [29,35,51]. Additional factors related to the denture geometry include the staircase effect [28,30]. Therefore, the object should be printed at an appropriate angle to control the mentioned factors and minimize errors.

#### 4.4.1. Support Structures

The building orientation affects the self-supporting geometry of an object [34]. As the printing orientation changes, the location of the supporting structure also changes [52]. In accordance with the principles of the 3D-printing technology, the produced item needs support when printing. It is recommended to avoid printing objects directly on the building platform without supporting structures, as this can lead to compression and projection of the initial layer near the platform due to additional laser exposure and can increase the overall thickness, especially in thin areas [10]. Moreover, each layer is printed on top of the preceding layer, resulting in a larger support area exposed to more UV light and shrinkage towards the supporting structure [31]. Therefore, the supporting structure, density, and position have an impact on the printing accuracy. When the printed part has many supporting structures, it shrinks to the side of the support structure as it is subjected to more UV exposure than the less dense areas of the support structure [27]. Owing to the support density, technical errors were reported with support removal (positive and negative deviation) especially when designed at the intaglio surface and affecting the accuracy. However, many additional supports, extra support, and bars are recommended to avoid overhang areas [28].

The trueness of intaglio surfaces can be significantly affected by the placement or elimination of the support structure [30]. Therefore, it is recommended to locate the support at the cameo surface, because higher trueness values are observed in denture bases with support and support struts on the cameo surface than those without struts [27,30,52]. In addition to the position of the support, the removal of the support structure and support struts affects the trueness, as it could result in the distortion in the area of support attachment [28,30,34]. Positive deviations were seen in the area with more support structures and on the opposite side of the support structure. In term of the support density and printing angle, the 90° build angle had the fewest support structures, followed by 45°, which in turn showed less support structure density than 0° [29,53]. Because of the large horizontal area printed in each layer, the 0° build angle group showed many support structures, further distorting the printed structure. In addition, the 0° group displayed inadequate denture base adaptation, particularly in the peripheral seal, posterior palatal seal, and primary bearing area [31]. When the material that has already polymerized can tolerate the overhang structure during the polymerization process, a small portion of an object can be printed without the need for supporting structures [34]. In layered manufacturing, building external supports is necessary to prevent the product from toppling or supporting floating components and overhanging materials [50]. The CD base with the highest fitting precision had a 45° building orientation and support structures on the CD base’s cameo surface and yielded better outcomes compared with other orientations [21,27,29,34,50,53]. This is in addition to the self-supporting geometry and less overhang structure with 45 degrees [21,27,29,34].

A supporting structure and distribution must be created for any area that requires external support to withstand overhangs. Inadequate support could cause 3D-printed objects to be distorted or inaccurate. With SLA and DLP printers, the supporting structures are made from the resin used for the printed object; consequently, the supporting structures should be designed and positioned to be easily removed [21,27,29,34,50]. Some supporting structures leave notches after removal, so the connection between the support and the printed resin should be as thin as feasible [21]. As this study focused on tissue side adaptability, the removal of the support structure on the cameo surface is of low concern as this surface will be polished, while the removal of supporting structures at the intaglio surface should be considered as this directly affects the tissue side adaptability. However some studies did not mention the details of support removal while others gently removed using a carbide bur [31] or using side cutters [32]. In addition, other studies [27,28,29,30,33,35,36] did not mention any details about support removal. Whatever the tool and method, it is difficult to remove the support structures without impairing the denture surface where the support is attached [16]. Therefore, it is recommended to design the support structure on the cameo surface and away from the intaglio surface and tooth socket. However, in MJP printers, wax materials are used as supporting structures, which are easily removed with a warm water wash and heating oven. Consequently, the ability to melt and clean the supporting structures without harming the surface of the 3D-printed product is one benefit of using an MJP printer [34].

In term of denture configuration, the build orientation affects the concave and convex areas of the denture base’s intaglio and cameo surfaces. For example, the unpolymerized monomer likely accumulates in the concave area of the denture base at the intaglio surface and denture tooth sockets, especially at 0° [35]. As the denture socket accuracy is affected, occlusion and tooth position can be affected in the case of removal of the support structure connected to the denture sockets. The support position and support removal process from the denture socket should be considered in clinical practice, as they can affect the occlusal relationship and adhesion of the denture teeth [29]. Alterations to the support structure location, printing sequence, and direction of AM could possibly have an impact on the dimensional accuracy. Also, the surface deviation of the DLP and SLA denture base may be influenced by the platform moving upward and the 3D printable material sagging compared with the MJP platform movement [27,34,35]. Based on study analysis, printing technology associated with different parameters affects the accuracy of the printed denture base. In an SLA study [28] and an MJP study [34], the accuracy is orientation-dependent and 45 degrees showed the highest accuracy. DLP showed variations in accuracy between no significant changes [27,31] and significant changes [30,32,33,35] with 45 degrees having a high accuracy followed by 90 degrees. These variations were attributed to the printing technology in which the direction of printing and platform position per printer varied: (1) designed at the top of the printer where the printed object is hung upside down on the platform (DLP and SLA) or (2) designed at the bottom of the printer with the printed object on the top of the plate (MJP) [29,36]. When comparing SLA and DLP according to the included studies, 45 degrees printed with SLA showed more accuracy than DLP [29]. In another study [36], SLA has a better trueness while SLA and DLP have the same precision and 90 degrees is recommended in terms of accuracy when both technologies are used. This is owing to the control of the light penetration depth through the monomer and light refraction, in addition to the reproducibility of SLA printing where the selected laser intensity and selected parameters are able to prevent light refraction [16]. Each technology has its own features and, owing to the variations in findings, further investigations are recommended for comparison between different printing technologies’ effect considering the printing orientation and anisotropic parameters affecting the accuracy as well as the strength of the printed objects.

#### 4.4.2. Starting Point and Separation Force Effect

The starting points of the printing are changed by the different build angle. The uneven form of the CD base results in many printing starting points when the support structure is designed to be parallel to the build platform (angles of 0° and 180°). Conversely, CD bases have fewer starting points at 45° and 225° angles. For example, a CD base with a build angle of 90° has two starting points and just one for an angle of 270° [35]. The printing accuracy varies with variable starting point numbers and positions, even when the model build angles are the same [29,51]. In addition, curling and warping phenomena contribute to dimensional inaccuracy, and these occur when a new layer is polymerized on the previously polymerized layer [54].

When printing starts from the labial or palatal side at 90°, negative and positive deviations are noticed in the palatal rugae. However, when printing from the buccal side, the left and right sides of the palate show negative and positive deviations, respectively [32]. This can be mainly explained based on the separation force effect which exists in bottom-up exposure DLP and SLA and top-down exposure MJP systems, resulting in deformation of the printed part [52]. The separation force can be affected by the printing systems and polymer properties [9,55]. Each base layer in the printing process adheres to the resin tank bottom after curing, and the cured layer is then pulled by platform movement using a certain separation force. At this phase, tensile tension is applied to the base along the *Z*-axis until it completely separates from the resin tank bottom. Therefore, the deviation distribution groups differ under these forces [9,55]. Despite efforts by researchers to reduce the separation force, the crucial issue has not been resolved. One potential solution is changing the printing parameters to minimize the separation force effect on the denture base [28,32].

#### 4.4.3. Polymerization Shrinkage

The locations of positive and negative deviations vary; for instance, positive deviations are primarily located in the maxillary residual ridges and tuberosities, while negative deviations are found in the palate. The contraction of centripetal polymerization during curing could be the cause of this phenomenon [16,36]. In order to complete the curing process, post-polymerization is necessary since a high photoinitiator concentration causes the photosensitive resin to cure rapidly when exposed to UV light and leaves large amounts of residual initiators behind after the initial curing [42]. The bases shrink during post-polymerization, which could possibly account for the greater deviation observed at the denture border as opposed to the palate or residual ridge [32].

#### 4.4.4. Orientation and Layer Number

The number of layers is related to the build orientation. In the supporting software, the reference dentures with varying build orientations are cut into varying numbers of layers, each with a constant thickness. For example, the denture with 90° angles has the most layers. The dimensional error of 3D-printed complete dentures increases with the number of layers [31,34]. Different build orientations cause variations in the denture base’s exposed shapes and the number of layers [33,34]. The variations arise from the unique characteristics among the 45° orientation group’s printing layers. The 3D-printed resins are linked stepwise, and the step edges between layers induce errors in dimensional accuracy [32,34]. According to research, this might be because as thickness decreases below a certain point, a part of the region deviates from each layer’s ideal boundary, and as the number of layers rises, the likelihood of probable errors increases [33].

#### 4.4.5. Staircase Effect

**Figure 3:**
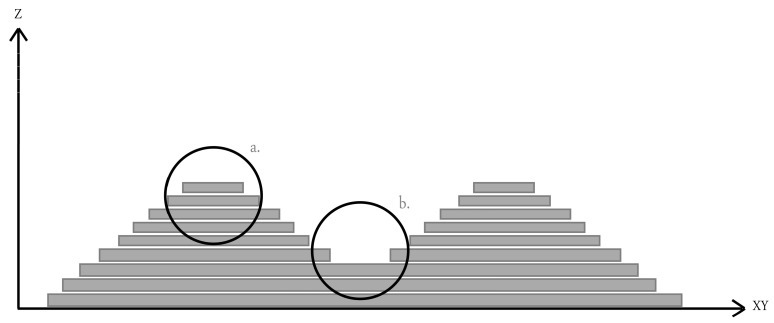
Illustrated figure for staircase effect. (a) Convex staircase effect; (b) Concave staircase effect.

Figure 3 illustrates the concept of the staircase effect and the relationship between the printing orientation and layer thickness. The cusp height is the highest variation brought about by the staircase effect between the CAD model surface and the printed layer surface. The variation in cusp height depends on the printing orientation, layer thickness, and angle (θ) formed by the normal CAD model surface. The cusp height decreased with a thin printed layer, and a small angle (θ) resulted in a high surface accuracy [28]. According to Charoenphol et al., the designed structure is built layer by layer during the 3D-printing process, producing the effect of a staircase. This effect can be seen on the surfaces due to the offset between layers in curved and oblique locations. When printing the structure on large, curved surfaces, more steps become visible, and the distance between two successive layers gets larger. The staircase effect can be minimized by optimizing the building angle [56]. In addition, it is best to place the object at an angle that allows for a gradual transition between two successive printing layers [31].

All printed bases have a surface structure that resembles stairs due to denture geometry. The staircase effect results in a reduction in printing resolution and has a direct relation with printing layer thickness [28]. It was expected that 50 µm would have a smaller staircase effect than 100 µm because of the increased angle (θ). Nonetheless, the 50 µm and 100 µm have a comparable accuracy. In a study by You et al. [16], a trial denture’s intaglio surface with a layer thickness of 100 µm was measured with an accuracy superior to 50 µm. Therefore, it is possible that the staircase effect has more impact on the surface characteristics than the denture bases’ manufacturing accuracy [32].

#### 4.4.6. Time and Material Consumption in Relation to Angle

The main consideration while producing different prostheses is cost-effectiveness. There is a significant time and material consumption associated with different printing orientations. At the 90° build angle, the denture occupies more space on the platform, allowing only a limited number of complete dentures to be printed simultaneously, with each denture requiring approximately 45 min of printing time. The denture base printed at a 45° build angle uses less space on the platform than the base printed at 0° and has a longer printing time of approximately 60 min. At 90°, the denture occupies the least space on the platform compared with other angles, allowing for multiple denture printing and requiring around 80 min printing time. With 90°, only a small number of supports are required. As the number of supports decreases, the material saving and the error related to support structures also decrease, as well as the time required for support removal [31]. Printing orientation has an impact on the printing time and material consumption, which should also be considered as key factors affecting the selection of printing strategies [32]. In addition to time and in terms of layer thickness, using a 50 µm layer thickness requires approximately 1.79 times more printing time than using a 100 µm layer thickness [32].

### 4.5. Printing Angles: Which Degree Is Better and Recommended?

Dentures printed at 45° showed the lowest RMSE values [28,30,31,32,33,34]. It was reported that when the printing angle surpassed 45°, overhang occurred in some areas, necessitating the addition of support structures to the object’s surface, which could adversely affect its surface accuracy [56]. The RMSE values for the trueness of dentures printed at 90° are larger than those printed at 45° [28,30]. Charoenphol et al. reported that denture bases printed at a build angle of 45° exhibit a better accuracy than those printed at 90° and 0° [31]. Though different DLP 3D printers and groupings were used in the previously mentioned studies, the 45–90° build orientation range yielded the most favorable results. According to the research of Song et al., build orientation influences manufacturing accuracy; 45° and 90° build orientations are advised based on their proven accuracy. Furthermore, the 45° build orientation should be utilized if rapid production is necessary [32]. According to Lee et al., the denture base exhibits the maximum trueness when created at 45°; the highest trueness is shown by the lowest RMSE value [33]. Jin et al. [27] recommend a build orientation of 135° (45°) based on the deviation distribution pattern in the color map. For mandibular CDs, the 45° build orientation group produced the most accurate 3D-printed mandibular CDs [34], while another study recommended a 270° build for fabricating mandibular denture bases using DLP [35].

### 4.6. Printing Angles: Which Degree Is Worst and Not Recommended?

The dentures printed at 0° showed the highest RMSE and displayed the least clinically acceptable fit compared with other printing orientations [28,30,57]. Based on the staircase effect, when a denture is printed at 0° with a constant printing layer thickness, a large cos (θ) value is generated in the oblique and curved areas such as the crest of the ridge, palate, and denture border and has an impact on denture adaptation [56]. As the cos (θ) increases, the accuracy and adaptation are negatively affected [28,30,58]. Additionally, 0° exhibits areas of deviation on the posterior palatal border affecting the posterior palatal seal and denture retention [30]. When the printing direction is 0°, a staircase effect is seen; however, staircase effects are less noticeable at the 45° and 90° build angles. As a result, compared to the 45° and 90° groups, the denture base printed with a 0° build angle shows inferior denture adaptation in the palatal seal, peripheral seal area, and primary bearing area [28,31].

### 4.7. Summary and Recommendations

CAD/CAM innovation and the wide distribution of materials and technologies has an impact on prosthetic treatment and clinical outcomes [59]. Many investigations have been performed on different aspects and different levels; however, further investigations are still required. Due to the low number of included studies and lack of quantitative meta-analysis, this review cannot focus on and claim one printing orientation as the same orientation in one study not demonstrating the same behavior in another study which can mainly be attributed to variations in the methodology. However, we can summarize the preferred printing orientation based on the finding of this review until further studies are conducted in the same aspect covering the area of this review. In summary, the ideal build orientation is 45°, followed by 90°, despite the different printers and denture base materials utilized in the included studies. This finding may be related to the unique geometry of the CD base. Better interactions are established between elements like polymerization shrinkage, support conditions, and the separation force effect, which would otherwise reduce the accuracy of the CD. The accuracy of CD bases produced in these build orientations is higher, and the deviation distribution patterns of these bases align better with the properties of movable edentulous tissues. As a result, improving the build orientation may help with denture retention, mucosal support, and pressure point reduction. Additional clinical research is necessary to confirm the association between the suggested build orientation with proper support conditions and clinical outcomes.

The low number of included studies and the variations between printing parameters between studies could be considered as a limitation of this review. Moreover, meta-analysis could not be conducted due the variations between the included studies which were mainly due to the different printing orientations, thermal aging, support structures and positions, and different printing materials and technologies. In addition, there is the limitation of in vitro studies, as all of those included were in vitro studies. Therefore, future clinical studies are recommended, especially with growing research in this area: 3D printing and the related parameters’ effects. Due the heterogeneity of the methodology and protocols of the included studies, a meta-analysis could not be conducted which is considered as another limitation of this systematic review. A further review with a high number of included studies of close methodology and protocols as well as high-evidence or clinical studies are recommended to cover the effect of orientation and different parameters on the accuracy of 3D-printed denture base resins.

## 5. Conclusions

Printing orientation affects denture accuracy and adaptability and must be considered along with other printing parameters. The support structure and printing sequences, which depend on the orientation, affect the accuracy of the 3D-printed denture bases. Changing the build angle leads to adjustments in the support density, positioning, and the starting points for printing. The support position has more effect than the support density, and supports positioned on the cameo surfaces result in a high accuracy. If more support is indicated, additional bars or struts can be added to the cameo surface only. The 45° build angle is recommended, followed by 90°, regarding denture base adaptation and accuracy. Both recommended angles considering printing technology and support structure conditions necessitate further investigations to prove the fit, adaptability, clinical outcome, and durability of printed denture bases.

## Figures and Tables

**Table 1:** Search strategy.

Study question	What is the effect of printing orientation on the accuracy and fit of a 3D-printed denture base?
Search combination	“denture base” OR “acrylic resin” OR “prosthesis” OR “dental prosthesis” OR “removable dental prostheses” OR “maxillary complete denture” OR “mandibular complete denture” AND “Three-dimensional printing” OR “printing orientation” OR “building direction” OR “build orientation” OR “printing angle” OR “build angle” OR “printing parameter” OR “3D print” OR “additive manufacturing” OR “rapid prototype” OR “CAD/CAM” OR “stereolithography” OR “digital light projection” OR “3D printing” AND “accuracy” OR “dimensional change” OR “trueness ” OR “precision” OR “adaptation” OR “fit” OR “fitting accuracy“
Database search	PubMed, Web of Science, Scopus

**Table 3:** Quality assessment and risk of bias considering the aspects reported in the Materials and Methods Section (Faggion 2012) [26].

Author/Year	Sample Size Calculation	Sample Randomization	Control Group	Stating Clear Testing Method	Statistical Analyses Carried Out	Reliable Analytical Methods	Blinding of Evaluators	Risk of Bias
Jin et al., 2018 [27]	Yes	No	No	Yes	Yes	Yes	No	Medium
Hada et al. 2020 [28]	Yes	No	No	Yes	Yes	Yes	No	Medium
Yoshidomea et al. 2021 [29]	No	No	Yes	Yes	Yes	Yes	No	Medium
Cameron et al., 2022 [30]	Yes	No	No	Yes	Yes	Yes	No	Medium
Charoenphol et al. 2022 [31]	Yes	No	No	Yes	Yes	Yes	No	Medium
Song et al. 2023 [32]	No	No	No	Yes	Yes	Yes	No	High
Lee et al. 2023 [33]	No	No	Yes	Yes	Yes	Yes	No	Medium
Gao et al. 2021 [34]	No	No	Yes	Yes	Yes	Yes	No	Medium
Chaiamornsup et al., 2023 [35]	No	No	No	Yes	Yes	Yes	No	High
Unkovskiy et al., 2021 [36]	No	No	Yes	Yes	Yes	Yes	No	Medium

A “yes” was assigned where the parameter was reported in the text, and a “no” if the information was absent or unclear. The risk of bias was classified according to the sum of “yes” marks received as follows: 1 to 3 = high, 4 to 5 = medium, 6 to 7 = low risk of bias.

**Table 4:** Mean values and SD of accuracy per measurement method.

Study/Denture	Measurement Methods/Unit	Orientation°	Trueness Mean ± SD	Precision Mean ± SD	+Ve Deviation	−Ve Deviations	Comment on Values Presentations
Jin et al., 2018 [27] *Maxillary and mandibular denture*	RMSE, PA, and NA (mm)	90	0.095 ± 0.008		0.061 ± 0.002	−0.083 ± 0.007	Maxillary
		100	0.079 ± 0.003		0.053 ± 0.002	−0.074 ± 0.002	
		135	0.087 ± 0.007		0.039 ± 0.004	−0.072 ± 0.004	
		150	0.088 ± 0.006		0.038 ± 0.002	−0.074 ± 0.006	
		90	0.114 ± 0.005		0.095 ± 0.003	−0.089 ± 0.006	Mandibular
		100	0.103 ± 0.007		0.090 ± 0.005	−0.073 ± 0.006	
		135	0.123 ± 0.008		0.105 ± 0.007	−0.082 ± 0.005	
		150	0.136 ± 0.015		0.097 ± 0.008	−0.102 ± 0.010	
Hada et al. 2020 [28] *Maxillary denture*	RMSE/mm	0	0.129 ± 0.006	0.072 ± 0.004	----	-----	Trueness values
		45	0.086 ± 0.004	0.050 ± 0.003	------	-----	
		90	0.109 ± 0.005	0.069 ± 0.002	-------	-------	
Yoshidomea et al. 2021 [29] *Maxillary denture*	RMS/mm	0					Results present in tables as the average and with no mean values and standard deviation.
		45					
		90					
		135					
		180					
		225					
		270					
		315					
Cameron et al., 2022 [30] *Maxillary denture*	RMS/µm	0	68.1 ± 4.2 µm		57.6 ± 3.1 µm	−55.5 ± 6.4 µm	
		15	74.8 ± 8.3 µm		62. ± 2 6.8 µm	−54.2 ± 4.4 µm	
		45	60.2 ± 3.9 µm		44.6 ±2.2 µm	−43.3 ± 6.1 µm	
		60	56.2 ± 7.2 µm		40.6 ± 7.7 µm	−42.5 ± 6.8 µm	
		90	58.6 ± 4.5 µm		37.7 ±3.4 µm	−45.8 ± 4.3 µm	
Charoenphol et al. 2022 [31] *Maxillary denture*	RMSE/mm Overall surface area	0	0.1209 ± 0.0033				Three readings: overall, peripheral and posterior palatal seal areas, and the primary bearing area
		45	0.1265 ± 0.0036				
		90	0.1219 ± 0.0037				
Song et al. 2023 [32] *Maxillary denture*	RMS/mm intaglio surface	0	0.095 ± 0.016				Accuracy palate, residual ridge, borders, and intaglio surface
		45 labial	0.076 ± 0.010				
		90 labial	0.078 ± 0.012				
		45 posterior	0.098 ± 0.016				
		90 posterior	0.120 ± 0.008				
		45 buccal	0.088 ± 0.009				
		90 buccal	0.129 ± 0.011				
Lee et al. 2023 [33] *Maxillary denture*	RMS/µm	0					High resin viscosity–Layer thickness 50 µm
		45					
		90					
		0					High resin viscosity–Layer thickness 100 µm
		45					
		90					
		0					Low resin viscosity–Layer thickness 50 µm
		45					
		90					
		0					Low resin viscosity–Layer thickness 100 µm
		45					
		90					
Gao et al. 2021 [34] *Mandibular denture*	RMS/mm Whole denture	0	0.185 ± 0.060				Whole denture, teeth, denture extension, intaglio surface
		45	0.170 ± 0.043				
		90	0.183 ± 0.044				
	RMS/mm Intaglio surface	0	0.228 ± 0.010				
		45	0.207 ± 0.006				
		90	0.218 ± 0.057				
Chaiamornsup et al., 2023 [35] *Mandibular denture*	RMS/mm	0					Results presented in a bar chart and with no mean values and standard deviation.
		45					
		90					
		135					
		180					
		225					
		270					
		315					
Unkovskiy et al., 2021 [36] *Maxillary denture*		0	0.094 ± 0.004	0.087 ± 0.042	0.082 ± 0.011	−0.054 ± 0.006	SLA
		45	0.132 ± 0.016	0.094 ± 0.034	0.099 ± 0.015	−0.089 ± 0.018	
		90	0.083 ± 0.009	0.098 ± 0.037	0.055 ± 0.009	−0.045 ± 0.010	
		0	0.256 ± 0.031	0.134 ± 0.028	0.166 ± 0.027	−0.187 ± 0.024	DLP
		45	0.211 ± 0.031	0.048 ± 0.023	0.101 ± 0.010	−0.097 ± 0.008	
		90	0.163 ± 0.030	0.044 ± 0.023	0.066 ± 0.010	−0.065 ± 0.006	

## Data Availability

The data are available upon request via email or phone to the corresponding author.

## Appendix A. Guidelines and Glossary Related to 3D Printing Technology and Printed Resins

**Terms**	**Definition, Description, and Explanation**
Color map [28,30,32,33,34,35,36]	Color range indicating the clinical relevant areas. Different color interpret color mapping; light green to green (nominal deviations, acceptable deviation). Areas beyond nominal deviations are categorized as positive (+ve, yellow to red) or negative (−ve, light blue to blue)
+ve deviations [28,30,32,35]	The fabricated denture base data were larger than the CAD data and exceeding the allowable range limit (0.3 mm) indicating a gap between denture and mucosa affecting denture stability and durability.
−ve deviations [28,30,32,35]	The fabricated denture base data smaller than the CAD data and exceeded the lower limit of the allowable range (−0.3 mm) indicating an intimate contact with pressure on the mucosa which necessitate adjustment in the intaglio surface of denture base.
Root mean square error (RMSe) [28,30,31,32,34]	The RMSe values are overall accuracy measurement method via superimposition of two virtual files. The RMSE value, which was close to zero, meant the good adaptation of the denture base.
Trueness [28,30]	Closeness of measured values to the true value. The trueness value increased when the printed object and the CAD-designed object were dimensionally close
Precision [28,30]	Closeness of measured values during repeated measurements. The precision value increased when the printed objects were dimensionally close
Anisotropic Wikipedia	Anisotropy (/ˌænaɪˈsɒtrəpi, ˌænɪ-/) is the structural property of non-uniformity in different directions. An anisotropic object has properties that differ according to direction of measurement.
Staircase [28]	In printed surface with curvatures, the angle (^θ^) between two 3D printed successive layers resulted in staircase effect and expressed as the cusp height (CH). Large CH which resulted from thick printing layer/large cos (^θ^) negatively affect surface accuracy. - Concave staircase effect: Positive deviation - Convex staircase effect: Negative deviation
overhang areas [34]	Areas of a 3D printed object are not supported by supporting structures
